# Complete Mitogenome Analysis of Five Leafhopper Species of Idiocerini (Hemiptera: Cicadellidae)

**DOI:** 10.3390/genes13112000

**Published:** 2022-11-01

**Authors:** Lili Tian, Wenxin Yang, Chengyan Si, Xianguang Guo, Bin Zhang

**Affiliations:** 1 College of Life Sciences & Technology, Inner Mongolia Normal University, Hohhot 010022, China; 2 Chengdu Institute of Biology, Chinese Academy of Sciences, Chengdu 610041, China; 3 University of Chinese Academy of Sciences, Beijing 100049, China

**Keywords:** mitochondrial genome, next-generation sequencing, Idiocerini, selective pressure, tandem repeats

## Abstract

Insect mitochondrial genomes (mitogenomes) are of great interest in exploring molecular evolution, phylogenetics, and biogeography. So far, only 12 mitogenomes of the leafhopper tribe Idiocerini have been released in GenBank, although the tribe comprises 488 known species including some agricultural, forestry, and horticultural pests. In order to compare and analyze the mitochondrial genome structure of Idiocerini and even the selective pressure of 13 protein-coding genes (PCGs) of the family Cicadellidae, the complete mitogenomes of five species including *Nabicerus dentimus*, *Sahlbergotettix salicicola*, *Podulmorinus opacus*, *Podulmorinus consimilis*, and a new species of a new genus were determined by next-generation sequencing. The size of the newly determined mitogenomes ranged from 14,733 bp to 15,044 bp, comprising the standard set of 13 PCGs, 22 transfer RNA genes, two ribosomal RNA genes, and a long non-coding control region (CR). The extent of purifying selection presented different pictures in the tribe and the family. The less pronounced genes (0.5 < dN/dS < 1) were *nad5* and *nad4l* in Idiocerin, whereas in the family Cicadellidae including the sequences of Idiocerin, *nad1*-*nad6* and *cox1* genes were less pronounced. The codon encoding leucine was the most common in all species, and the codon encoding serine 1 was the most common in all species except for *P. opacus*. Interestingly, in *P. opacus*, another of the most common codons is that encoding serine 2. Among the 17 examined species of the Idiocerini, 14 species contained the tandem repeats, and 11 species of them contained the motif “TTATA”. These findings will promote research on the structure and evolution of the mitochondrial genome and highlight the need for more mitogenomes in Cicadellidae.

## 1. Introduction

The mitochondrial genome (mitogenome) is increasingly popular in various scientific disciplines such as animal comparative genomics, molecular evolution, phylogenetics, and biogeography [1,2,3,4]. In comparison with its individual genes widely used in phylogenetics and biogeography, the mitogenome can be more phylogeny-informative and provide multiple genome-level characteristics such as gene order and rearrangements, RNA secondary structures, and modes of control of replication and transcription [5,6,7,8,9]. The mitogenome of insects is a small double-stranded circular molecule of 14–20 kb in size, containing 37 genes including 13 protein-coding genes (PCGs), 22 transfer RNA genes (tRNA), and two ribosomal RNA genes (12S and 16S rRNA) [5,9]. Additionally, it contains a A + T-rich control region that plays a vital role in the initiation of transcription and replication [10].

Eurymelinae is one of the largest subfamily in the family Cicadellidae (typical leafhoppers). This subfamily, with a cosmopolitan distribution, comprises more than 1400 species in 182 genera of 11 tribes (https://www.catalogueoflife.org/data/taxon/8JK49 (accessed on 14 October 2022)). Species of the subfamily feed on a variety of trees and shrubs including several of economic importance, with some considered agricultural pests [11]. Recently, based on phylogenetic analyses with molecular and morphological data, the expanded concept of Eurymelinae [12] has been confirmed and six more monophyletic groups have been recognized as new tribes and the status of the Idiocerini tribe was redefined by Xue et al. [13].

As such, the tribe Idiocerini, which was earlier placed as the subfamily Idiocerniae of the family Cicadellidae, is now confirmed as one of the tribes in the subfamily Eurymelinae [13]. Currently, in the tribe Idiocerini, 68 genera represented by 488 species have been described (https://www.catalogueoflife.org/data/taxon/8JKKB (accessed on 14 October 2022).

Despite the advancement of sequencing technology including next-generation sequencing, the complete mitochondrial genome of species from the family Cicadellidae was less than 90, and only about 12 of the tribe Idiocerini have been released in GenBank (as of April 2022). Additionally, only one or two sequences per genera have been reported due to the limited research and sampling difficulty. Furthermore, the utilization of existing data of the genus *Idiocerus*, and even the tribe Idiocerini, has mainly focused on the announcement of mitogenome organization [14,15]. To date, little information is available for the systematic research on the mitochondrial genome structure of the tribe Idiocerini, and even of the family Cicadellidae.

In this study, we sequenced five complete mitogenomes of the tribe Idiocerini, which made up for the fact that few complete mitogenomes of this tribe have been reported (the previous five mitogenomes were announced by the team of Bin Zhang). The addition of five mitogenomes made it plausible to examine the mitochondrial genome structures at the level of tribe and even family, and to find several rules of these sequences including the selective pressure, tandem repeats of the control region, and other aspects. This study will promote the research on the structure and evolution of mitochondrial genomes, and highlight the need for more mitogenomes in the tribe Idiocerini and even of the family Cicadellidae.

## 2. Materials and Methods

### 2.1. Sample Collection and DNA Extraction

Five specimens, which was sequenced and analyzed in this study, belonged to five species in the tribe Idiocerini—*N. dentimus* Xue & Zhang, 2014, *S. salicicola* (Flor, 1861), *P. opacus* (Anufriev, 1978), *P. consimilis* (Vilbaste, 1968), and a new species of a new genus. Among them, the new species represents an undescribed new genus. The sequence of *Neoamritatus* sp. has been registered in GenBank under the genus *Neoamritatus* gen. nov., which has not been described yet [16]. The specimens were captured by sweep net in Inner Mongolia, Anhui, Zhejiang, and Yunnan Provinces from 2019 to 2021, and the specific information of sample collection is shown in Table 1. The captured specimens were fixed with 95% ethanol. The voucher specimens were preserved in 95% ethanol at −20 °C, and deposited at Inner Mongolia Normal University. The entire body of these five species without abdomen were shipped to Tsingke (Beijing, China). The total genomic DNA was extracted by following the method of CTAB [17].

### 2.2. Sequencing and Genome Assembly

The sample genome DNA was fragmented by mechanical interruption (ultrasound). Selected fragments were purified and repaired. The addition of A on the 3 end and the connection of sequencing connectors were completed. Fragment sizes (350 bp) were selected by the method of agarose gel electrophoresis, and PCR amplification was carried out to produce sequencing libraries, which were formed by the standard procedure of Illumina DNA library construction. NEBNext^®^Ultra™ DNA Library Prep Kit for Illumina^®^ was used to construct the libraries. The libraries were sequenced using Illumina Nova Seq platform (Illumina, San Diego, CA, USA). SOAPnuke v1.3.0 [18] was used to filter the raw data according to several standards including phred quality <Q5, N base number >5. All clean data were assembled by SPAdes v3.13.0 [19].

### 2.3. Annotation and Analysis

The online software of BankIt [20] was used to submit the complete mitogenomes to GenBank. NCBI BLAST [21] and MITOS [22,23] were used to identify the boundaries of PCGs and rRNAs. The potential cloverleaf structures and boundaries of tRNAs were identified by the online software tRNA scan-SE [24,25]. The mitogenomic map was generated by the online software OGDRAW v1.3.1 [26]. Non-synonymous and synonymous substitutions of PCGs of Idiocerini and Cicadellidae and nucleotide composition were computed in the software MEGA v7.0 [27]. Taxon information of 17 species of Idiocerini and the GenBank accession numbers are listed in Table 2. Composition skew values were computed by utilizing the formulae: AT-skew = ((A% − T%)/(A% + T%)); GC-skew = ((G% − C%)/(G% + C%)). The boundaries and the size of CR were confirmed by the position of *tRNA^Phe^* and *tRNA^Pro^*. In addition, the sequence comparison with previously reported Idiocerini mitogenomes is another significant method. Furthermore, the base distribution and relative synonymous codon usage (RSCU) values were calculated in MEGA v7.0. Tandem repeats in the CR were detected in the tandem repeats finder online server [28,29], and the results generated by the server were selected according to the copy number.

### 2.4. Relationships with the Mitogenomes of Published Idiocerini

Phylogenetic trees were reconstructed by using Bayesian inference (BI) and maximum likelihood (ML) methods for assessing the authenticity of the sequenced five leafhopper mitogenomes and their phylogenetic placements. Aside from the five mitogenomes of Idiocerini determined in this study, ten Eurymelinae mitogenomes and three outgroup taxa were downloaded from GenBank [41] (see Table 2). It is worth explaining that there are 12 sequences of Idiocerini released in GenBank, but we only retrieved 10 of them to analyze the relationships with the mitogenomes of the published Idiocerini, as there seem to be controversial perspectives on the species identification of *Idioscopus* sp. And *Rhytidodus viridiflavus*. However, we chose all of them to analyze the selective pressure and control region.

Based on the most recent knowledge on higher-level relationships of Cicadellidae [38,39], two subfamilies, Iassinae and Megophthalminae, are closely related to Eurymelinae (see Table 2). Accordingly, *B. lateprocessus* and *D. nigropicta* (available in GenBank) were selected as the outgroups. Furthermore, one mitogenome representing the subfamily Typhlocybinae, *L. lingchuanensis*, available in GenBank (accession number MN605256), was chosen to root the tree due to its relatively far related to Eurymelinae [38,39]. Concatenation of 13 PCGs and the alignment of these 18 sequences were processed in MEGA with the default parameters, and then we checked them manually. With the help of the plug-in program in PhyloSuite v1.1.16 [42], we completed gene partitioning and tree construction. Meanwhile, we selected the best partitioning schemes and evolutionary models, which were estimated by PartitionFinder v2.1.1 [43], with the greedy algorithm and corrected Akaike information criterion (AICc). In order to find the partitioning scheme models for ML and BI analyses, respectively, we utilized the “all” and “Mrbayes” modes. MrBayes v3.2.6 [44,45] was utilized for partitioned Bayesian analyses, with four independent runs for two million generations and sampling every 100 generations. Convergence of the runs was assessed by checking the likelihood scores of all trees on Tracer v1.7 [46] and the average standard deviation of split frequencies <0.01. The 50% majority-rule consensus tree and the posterior probability (PP) of clades were assessed by combining the sampled trees from the two independent runs after a 25% burn-in phase. We interpreted PP ≥0.95 to be strongly supported [47,48]. The information of the best-fit substitution models and partitioning schemes for PCGs are listed in Table S1. IQ-TREE v1.6.7 [49] was used to construct the maximum likelihood (ML) phylogenetic tree. We used an ultrafast bootstrap approximation approach with 5000 bootstraps. Nodes with UFBoot ≥95 were considered to be well-supported [50]. In the end, FigTree v1.4.3 [51] was used for the tree visualization and PowerPoint was used for the tree edit.

## 3. Results

### 3.1. Genome Organization and Base Composition

**Figure 1:**
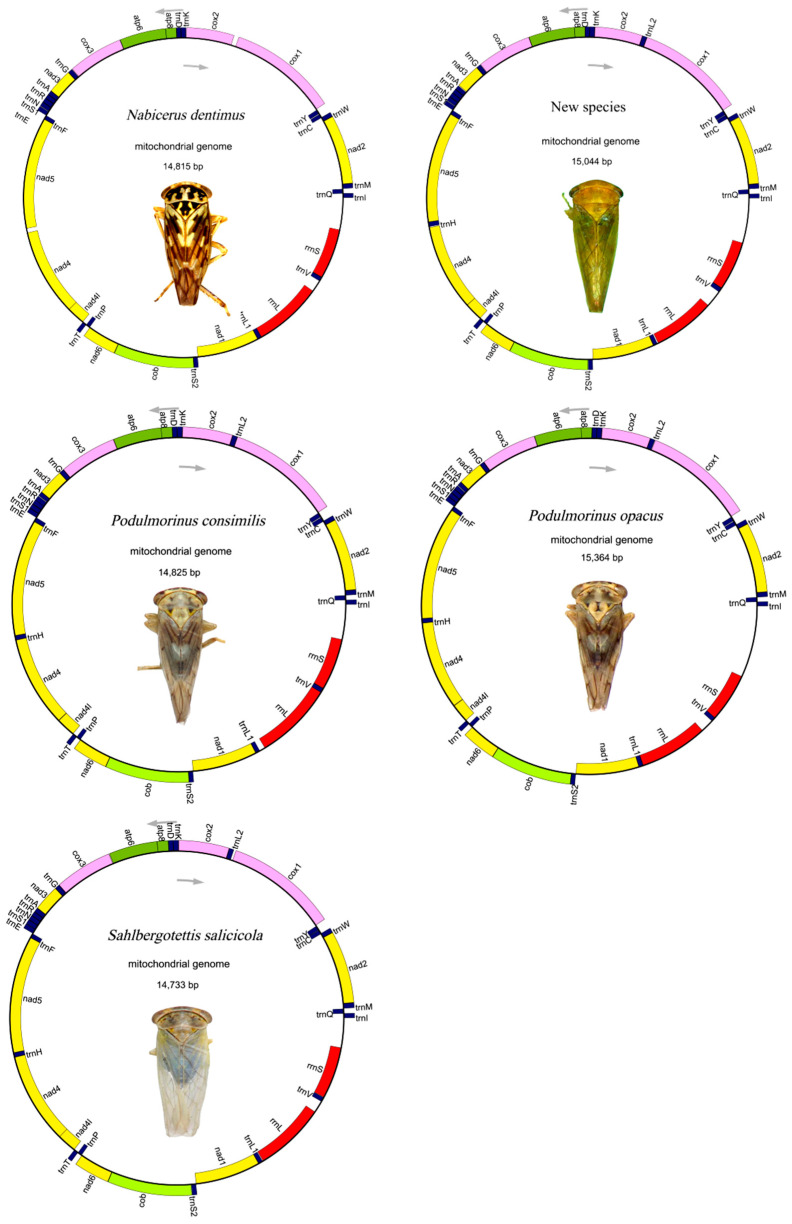
Mitochondrial genome diagram of five Idiocerini species. Genes encoded by the light strand are indicated inside or outside, respectively, showing the direction of transcription. The tRNAs are denoted in blue and labeled according to the one letter amino acid codes.

The lengths of mitogenomes of *N. dentimus*, *S. salicicola*, *P. opacus*, *P. consimilis*, and the new species were 14,815 bp, 14,733 bp, 14,815 bp, 14,825 bp, and 15,044 bp, respectively. The composition and arrangement of mitochondrial genes in these five species were the same as those in most other typical invertebrates (Figure 1, Table S2). They all consisted of 13 PCGs (*nad1*-*nad6*, *nad4l*, *atp8*, *atp6*, *cob*, *cox1*-*cox3*), 22 tRNA genes, two rRNA genes (12S rRNA and 16S rRNA), and one non-coding region (control region, CR).

The nucleotide composition, AT skew and GC skew of the total mitogenomes and PCGs of 17 species in the tribe Idiocerini were calculated. The mean AT nucleotide content of the five complete Idiocerini mitogenomes was nearly similar: 76.8% in *P. opacus*, 76.9% in *N. dentimus* and *P. consimilis*, 77.7% in the new species, and 79.1% in *S. salicicola*. Additionally, the nucleotide and composition skew values were conserved in Idiocerini (Table S2); they all showed a positive AT-skew (0.06 to 0.16), and a negative GC-skew (−0.44 to −0.05), suggesting a strong AT bias, and the AT content was lower in the PCGs than the total mitogenomes (from 75.1% to 77.9%).

### 3.2. PCGs and Codon Usage

All newly sequenced Idiocerini mitogenomes contained 13 protein-coding genes that ranged from 153 bp (*atp8*) to 1672 bp (*nad5*). The total length of the PCGs of these five species ranged from 14,733 bp (*S. salicicola*) to 15,044 bp (new species). Nine genes (*nad2*, *nad3*, *nad6*, *atp8*, *atp6*, *cob, cox1*-*cox3*) of the PCGs of these five mitogenomes were on the heavy strand, and the remaining four genes (*nad1*, *nad4*, *nad4l*, *nad5*) were on the light strand. There were five typical types of start codons (ATA, ATC, ATG, ATT, TGG), three typical types of stop codons that contained two truncated stop codons (T--, TA-) and one canonical (TAA), and the special stop codons (TAG), which were only presented in the *nad3* genes of *S. salicicola*.

**Figure 2:**
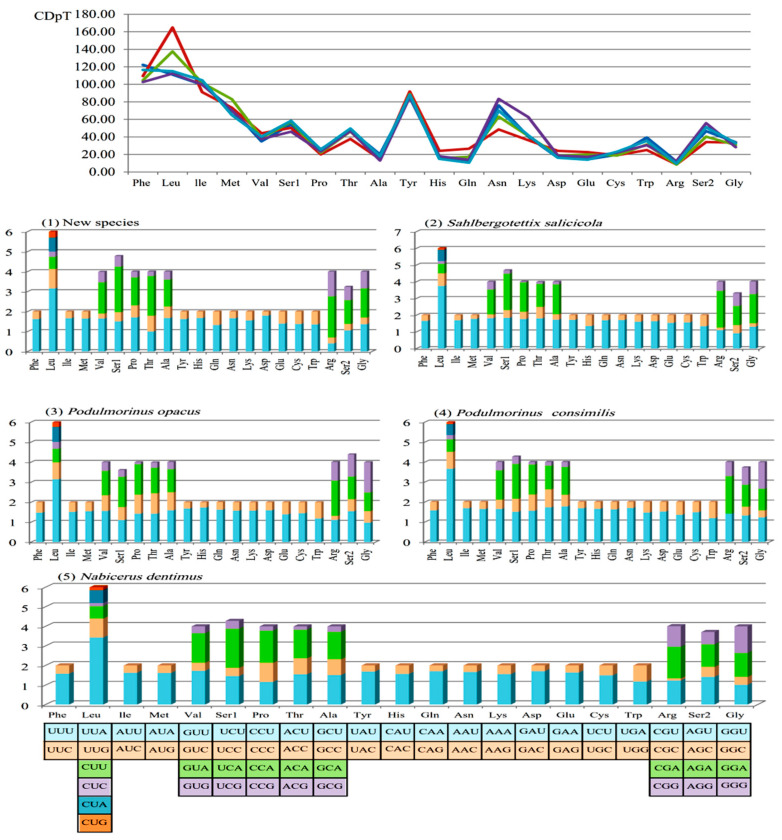
The base composition and the relative synonymous codon usage (RSCU) values (Y axes) of *N. dentimus*, *S. salicicola*, *P. opacus*, *P. consimilis,* and the new species, respectively. CDpT denotes codons per thousand codons.

The relative synonymous codon usage (RSCU) and codon distribution of these five Idiocerini mitogenomes were analyzed (Figure 2). The total number of these five species was similar: 3648 in *N.* *dentimus*, 3635 in the new species of the new genus, 3640 in *S. salicicola*, and 3648 in *P. opacus* and *P. consimilis*. The codon distribution among the four species were coincident. The codons encoding leucine and serine 1 were the two most frequently present. However, leucine and serine 2 were the two most frequently present in *P. opacusis*. The codons were biased to utilize more A/U than G/C at the end, which resulted in the content of AT being higher than GC in the third position of the Idiocerini PCGs.

### 3.3. Transfer RNAs and Ribosomal RNAs

The tRNA secondary structure and strand bias were coincident among these five Idiocerini species and even in other Hemiptera species [9,52]. Among the 22 tRNA genes, only *tRNA^s^^er^* (Ser1) could not be folded into a typical cloverleaf secondary structure and had no recognizable DHU arm (Figure S2). The length of the single tRNA gene varied from 61 bp (*tRNA^Arg^* in *S. salicicolaand*) to 72 bp (*tRNA^Lys^* in the new species of the new genus). Eight tRNAs were encoded on the light strand and the remaining were encoded on the heavy strand.

The 12S rRNA and 16S rRNA were located between the *tRNA^Leu^* (Leu1) gene and control region and interposed by the *tRNA^Val^* gene. The length of the 12S rRNA of these five species ranged from 741 bp (*S. salicicola*) to 754 bp (the new species of the new genus and *P. opacus*). The length of 16S rRNA ranged from 1012 bp (*S. salicicola*) to 1239 bp (*P. consimilis*). Additionally, the 12S rRNA and 16S rRNA of these five species were encoded on the light strand.

### 3.4. Non-Synonymous and Synonymous Substitutions

**Figure 3:**
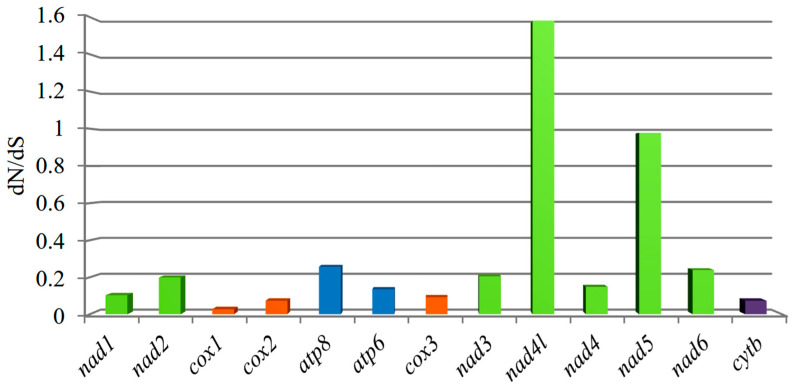
The nonsynonymous/synonymous ratios (dN/dS) in 13 mitochondrial PCGs of 17 species in Idiocerini. The histogram represents the average dN/dS for each PCG.

**Figure 4:**
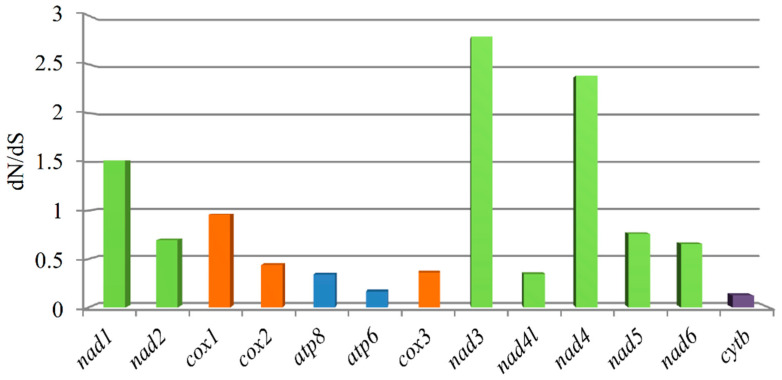
The nonsynonymous/synonymous ratios (dN/dS) in 13 mitochondrial PCGs of 167 species in Cicadellidae. The histogram represents the average dN/dS for each PCG.

To further understand the role of selective pressure and the evolution of PCGs, we computed the average dN/dS value of each PCG of 15 species in Idiocerini (Figure 3). Next, we also estimated the average dN/dS value of each PCG of 167 species in Cicadellidae (Figure 4), among which 162 complete mitogenomes were retrieved from GenBank (Table S3). The values of all PCGs except *nad4l* in Idiocerini were smaller than 1, which can be interpreted as meaning that the proteins evolve slowly under purify selection (i.e., are more conserved). Likewise, the values of all PCGs except *nad1*, *nad3*, and *nad4* in Cicadellidae were smaller than 1. This may be explained by the rationale that most of the nonsynonymous substitutions are detrimental to fitness and consequently have low fixation probabilities. Furthermore, the extent of purifying selection was less pronounced for *nad2*, *nad5*, *nad6*, and *cox1* in Cicadellidae. Meanwhile, only *nad5* for Idiocerini in comparison with the rest of the PCGs presented a less pronounced purifying selection. The *cox1* gene showed the lowest value (0.027), and *nad4l* showed the highest value (1.581) in Idiocerini. With the ratio dN/dS >1, *nad4l* of Idiocerini and *nad1*, *nad3*, *nad4* of Cicadellidae may be considered under positive selection.

### 3.5. Control Region

**Figure 5:**
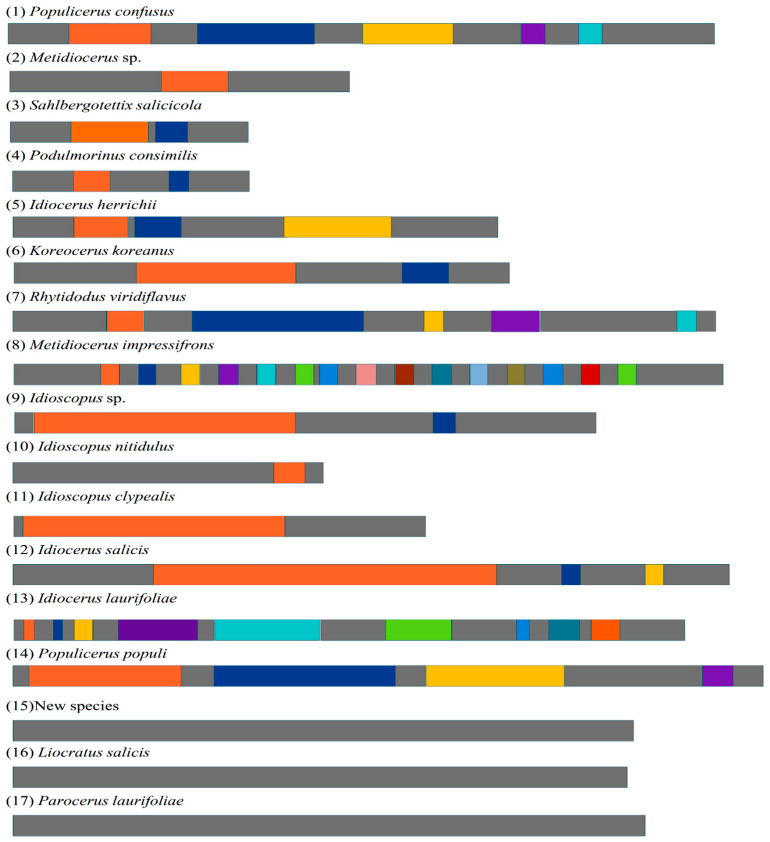
Types of the control region in Idiocerini, in consideration of the positions and related length of tandem repeats. Light black means the position without tandem repeats, other colors mean different tandem repeats of different species. The difference in color is just for presenting different tandem repeats of each species, and the same color of different species does not mean the same tandem repeats of them. Due to the space limit and more TRs of several species, the length of different tandem repeats was defined according to the total length of the control region of each species, and the total length of the control region was not mutually referenced.

Tandem repeats are one of the factors accountable for the extensive size variations in the mitogenomes [53,54]. In this study, we analyzed the tandem repeats of the 177 Idiocerini species. We found that 14 species had TRs, and 11 species had the motif “TTATA”. *M. impressifrons* contained the largest number of TRs of Idiocerini, which was 15. The length of the TR of *Idioscopus* sp. (218 bp) was the longest, and the length of the TR of *R. viridiflavus* (9 bp) was the shortest (Figure 5, Table S4). It is rather surprising that the control region of the new species of the new genus, *L. salicis* and *P.* *laurifoliae* had no TRs, which again proves that tandem repeats are one of the main reasons for the extensive variation in the length of CRs.

### 3.6. Relationships with the Mitogenomes of Published Idiocerini

**Figure 6:**
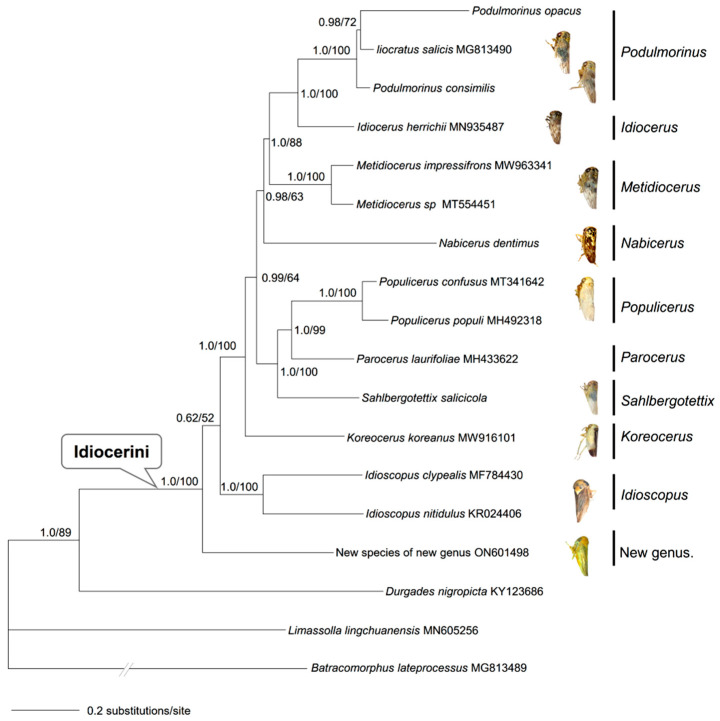
A 50% majority-rule consensus tree of the tribe Idiocerini inferred from the partitioned Bayesian analyses based on the concatenated 13 PCGs of 15 species and three outgroup taxa. Node numbers indicate Bayesian posterior probabilities and ML UFBoot values, respectively. Branch lengths represent the means of the posterior distribution. Genus/tribe assignments are listed.

The relationships with the mitogenomes of the published Idiocerini are shown in Figure 6. Bayesian inference and ML analyses produced an identical topology. Thus, only the BI tree with both PP and UFBoot from ML was presented. Monophyly of the tribe Idiocerini was recovered with strong support (PP = 1.0; UFBoot = 100). As can be seen from Figure 5, the placement of *Idioscopus* or the new genus was unresolved due to very low support (PP = 0.62; UFBoot = 52). *Koreocerus* is sister to a clade of the remaining taxa except for *Idioscopus* and the new genus. *Parocerus* was more closely related to *Populicerus* than to *Sahlbergotettix* (PP = 1.0; UFBoot = 99). Interestingly, monophyly of the genus *Podulmorinus* was challenged due to *L. salicis* being nested between *P. opacus* and *P. consimillis*. Overall, the intergeneric relationships in Idiocerini were tentative due to insufficient data in this study.

## 4. Discussion

In this study, we presented a comprehensive comparative analysis of mitogenome structures in Idiocerini and even in Cicadellidae including the comparison of the selective pressure of Idiocerini and Cicadellidae, and the comparison of the control region of Idiocerini. The extent of purifying selection presented different scenarios in the tribe and family; the *nad5* gene was the only gene showing the consistent pattern of the lower extent of purifying selection. Overall, the synonymous substitutions in the mitochondrial protein-coding genes evolved in a near neutral manner, whereas the pattern for nonsynonymous substitutions was mostly consistent with strong purifying selection [55]. Several genes show possible evidence for positive selection including the *nad4l* gene of Idiocerini and *nad1*, *nad3*, *nad4* genes of Cicadellidae. NADH dehydrogenase genes harbored an exceptionally high percentage of the total amino acid changes and showed a higher dN/dS compared to the other genes of mitochondrial genomes in Cicadellidae. A possible reason is that relaxed purifying selection is driving the evolution of *nad5* by mostly affecting regions that have lower functional relevance [55,56]. The phenomenon of the purifying selection of PCGs is usually detected in most Metazoa [53]. Thus, whether NADH dehydrogenase genes in Cicadellidae undergo positive or purifying selection requires further validation by other methods including physicochemical changes, the codon-based test, and so on. Further research is necessary to detect the variation in selective pressures among different Cicadellidae lineages, and to quantify the probability of positive selection on each site in each gene across all Cicadellidae species.

The tandem repeats of the control region of mitgenomes in Idiocerini were also systematically compared. Variable types were presented in this tribe, and no apparent rule was observed. Variable CR types could reflect the complicated evolution of the CR. To decipher the evolutionary processes that drive diversification in the CRs of Cicadellidae, further study is necessary to investigate the dynamics of CRs based on comparative methods in an explicit phylogenetic framework. Furthermore, a more detailed analysis of the tandem repeats would be interesting, as there is a great deal of variation. Thus, more CR sequences of Hemiptera and even of Auchenorrhyncha are needed for further study. Due to the very limited mitogenome data available in the tribe Idiocerini, the inferred phylogenetic relationship in Idiocerini is very tentative. The mitochondrial control region had not been considered as a transcriptional region until 2018, when Gao et al. [57] documented that this region encoded two long non-coding RNAs (lncRNAs). However, current methods on the annotation of animal mitogenomes is still limited to blastx or structure-based covariance models [21]. Thus, it is necessary to further use a small RNA sequencing (sRNA-Seq) based method [58,59] to obtain improved annotations of the insect mitogenome at 1 bp resolution and to decipher TRs in the CR.

## 5. Conclusions

We comprehensively compared the complete mitochondrial genomes of five species in the tribe Idiocerini including *N. dentimus*, *S. salicicola*, *P. opacus*, *P. consimilis*, and an undescribed species of an under described genus for the first time. In Idiocerini, the extent of purifying selection was less pronounced for *nad5* in comparison with the rest of the PCGs excluding *nad4l*. In Cicadellidae, the extent of purifying selection was less pronounced for the *nad2*, *nad5*, *nad6*, and *cox1* genes in comparison with the rest of the PCGs excluding the *nad1*, *nad3,* and *nad4* genes. The *nad5* gene of both the tribe Idiocerini and the family Cicadellidae was presented as 0.5 < dN/dS < 1. The NADH dehydrogenase genes in Cicadellidae exhibited variable features. Furthermore, the secondary structures of tRNAs were predicted. Moreover, the tandem repeats of the control region from 17 species were systematically analyzed for the first time; the motif “TTATA” was shared in 11 species. Meanwhile, a mitogenomic perspective on the phylogenetic relationship of the tribe Idiocerini was inferred for the first time. Overall, our results enrich our understanding of the structure of mitochondrial genomes in Idiocerini. More mitogenomes from different taxonomic groups in the Cicadellidae are needed to better understand their phylogenetic relationships.

## Figures and Tables

**Table 1:** List of the collection information for five species in the tribe Idiocerini.

Species	Family	Subfamily	Collection Date	Coordinates	Collection Site
*N. dentimus* Xue & Zhang, 2014	Cicadellidae	Eurymelinae	June 2020	30.08293° N, 118.14638° E	Tangkou Town, Huangshan District, Anhui Province, China
New species of a new genus	Cicadellidae	Eurymelinae	July 2020	27.37167° N, 119.76870° E	Sankui Town, Taishun County, Zhejiang Province, China
*S. salicicola* (Flor, 1861)	Cicadellidae	Eurymelinae	July 2020	40.43336° N, 111.96360° E	Shengle Town, Ringer County, Inner Mongolia Autonomous Region, China
*P. opacus* (Anufriev, 1978)	Cicadellidae	Eurymelinae	June 2020	24.97987° N, 102.28578° E	Dashiao Town, Luping County, Yunnan Province, China
*P. consimilis* (Vilbaste, 1968)	Cicadellidae	Eurymelinae	July 2020	27.78046° N, 119.79011° E	Huangqiao Protection Station, Wuyanling National Nature Reserve, Taishun County, Zhejiang Province, China

**Table 2:** Taxon information and the GenBank accession numbers of 20 species.

Taxon	Family	Subfamily	Accession Number	Length (bp)	Reference
*N. dentimus* (Xue & Zhang, 2014)	Cicadellidae	Eurymelinae	ON601496	14,815	This study
New species of a new genus	Cicadellidae	Eurymelinae	ON601498	15,044	This study
*S. salicicola* (Flor, 1861)	Cicadellidae	Eurymelinae	ON510775	14,733	This study
*P. opacus* (Anufriev, 1978)	Cicadellidae	Eurymelinae	ON601497	14,815	This study
*P. consimilis* (Vilbaste, 1968)	Cicadellidae	Eurymelinae	ON510776	14,825	This study
*Liocratus salicis*	Cicadellidae	Eurymelinae	MG813490	16,436	[30]
*Parocerus* *laurifoliae*	Cicadellidae	Eurymelinae	MH433622	16,811	[30]
*Populicerus* *confusus*	Cicadellidae	Eurymelinae	MT341642	16,395	[31]
*Populicerus* *populi*	Cicadellidae	Eurymelinae	MH492318	16,494	[30]
*Idioscopus* *nitidulus*	Cicadellidae	Eurymelinae	KR024406	15,287	[32]
*Idioscopus* *clypealis*	Cicadellidae	Eurymelinae	MF784430	15,393	[33]
*Idiocerus herrichii*	Cicadellidae	Eurymelinae	MN935487	15,489	[34]
*Idioscopus* sp.	Cicadellidae	Eurymelinae	MH492317	15,423	[30]
*Koreocerus koreanus*	Cicadellidae	Eurymelinae	MW916101	15,594	Liu’s unpublished data
*Metidiocerus* sp.	Cicadellidae	Eurymelinae	MT554451	15,079	[35]
*Metidiocerus impressifrons*	Cicadellidae	Eurymelinae	MW963341	16,426	[36]
*Rhytidodus viridiflavus*	Cicadellidae	Eurymelinae	MN935488	16,842	[37]
Outgroup					
*Batracomorphus lateprocessus*	Cicadellidae	Iassinae	MG813489	15,356	[38]
*Limassolla lingchuanensis*	Cicadellidae	Typhlocybinae	MN605256	15,716	[39]
*Durgades nigropicta*	Cicadellidae	Megophthalminae	KY123686	15,974	[40]

## Data Availability

Illumina raw reads were deposited in the NCBI Sequence Read Archive (SRA): *N. dentimus* in the BioProject SAMN27734787; *Neoamritatus* sp. in the BioProject SAMN27736168; *S. salicicola* in the BioProject SAMN27736201; *P. opacus* in the BioProject SAMN27736252; *P. consimilis* in the BioProject SAMN27736288. The mitochondrial genomes were deposited at GenBank with accession numbers ON601496, ON601498, ON510775, ON601497, and ON510776.

## Supplementary Materials

The following supporting information can be downloaded at https://www.mdpi.com/article/10.3390/genes13112000/s1. Figure S1: Putative secondary structures of the tRNAs identified in the mitogenomes of (a) *N. dentimus*, (b) new species of new genus, (c) *S. salicicola*, (d) *P. opacus*, and (e) *P. consimilis.* Figure S2: Maximum likelihood tree inferred from 13 concatenated PCGs. Node numbers represent ultrafast bootstrap support. GenBank accession numbers are given with species names. Table S1: Organization of mitochondrial genomes of the tribe Idiocerini. Table S2: Best-fit models and partitioning schemes selected by Partition Finder 2. Table S3: List of complete mitogenomes of leafhoppers in the family Cicadellidae retrieved from GenBank. Table S4: Tandem repeats in the mitochondrial control region of 14 species in the tribe Idiocerini.
