# Supplementary material for: Complete Mitogenome Analysis of Five Leafhopper Species of Idiocerini (Hemiptera: Cicadellidae)
Source: Genes (Basel). 2022 Nov 1;13(11):2000. doi: 10.3390/genes13112000 (PMC9690763; doi:10.3390/genes13112000)

(a) *Nabicerus dentimus*

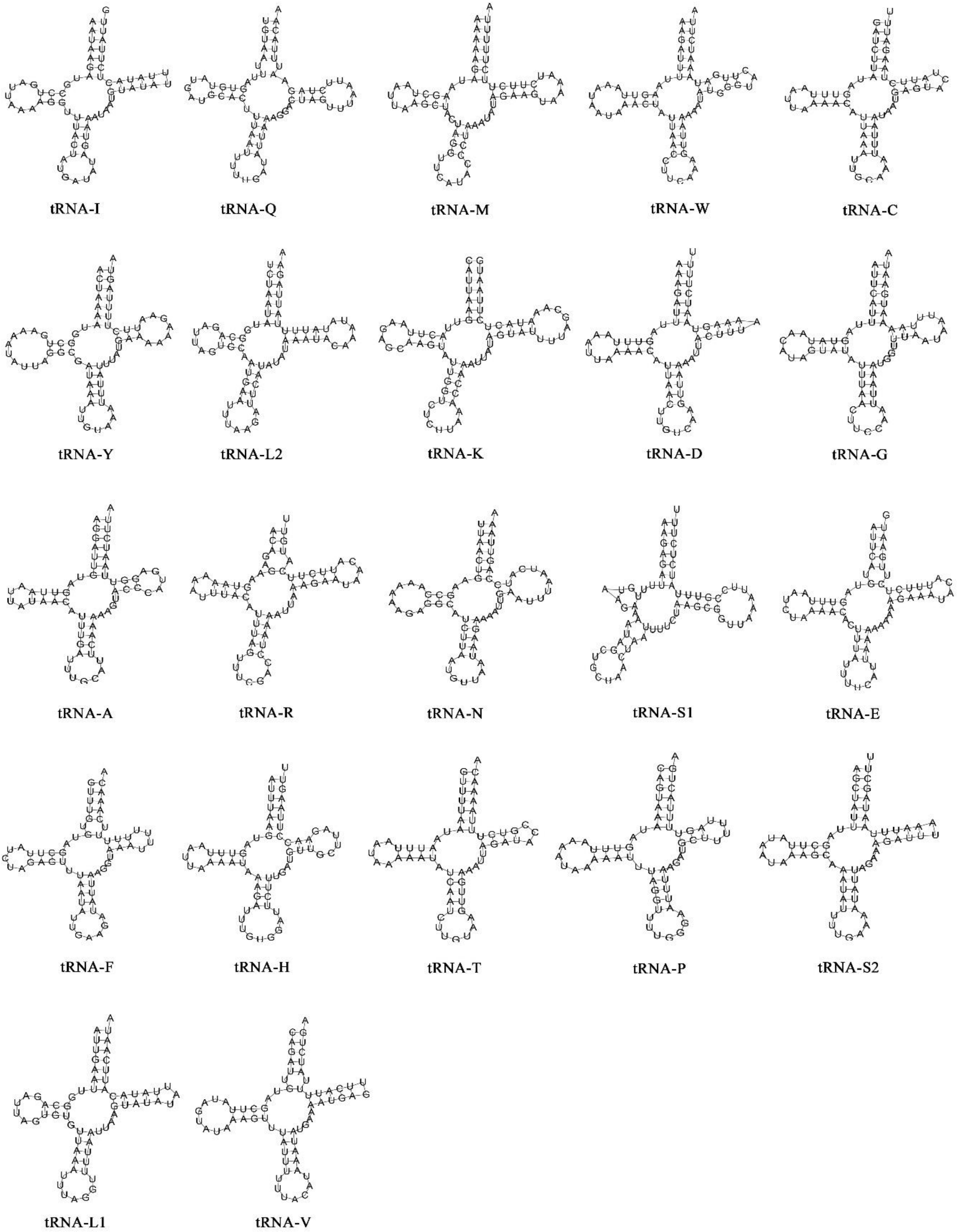

(b) *Neoamritatus* sp.

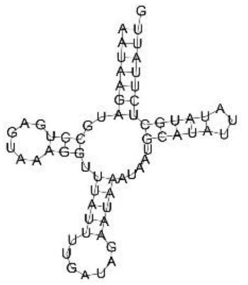

tRNA-I

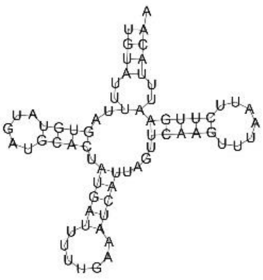

tRNA-Q

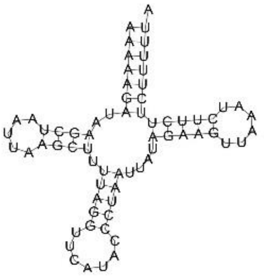

tRNA-M

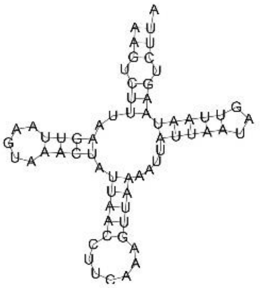

tRNA-W

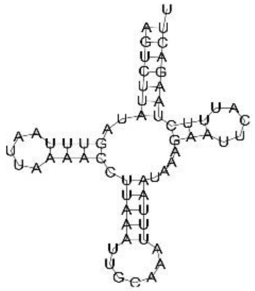

tRNA-C

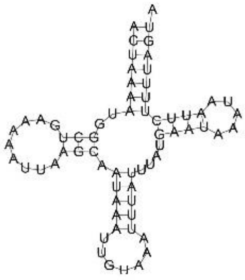

tRNA-Y

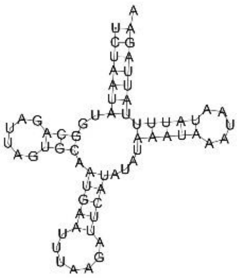

tRNA-L2

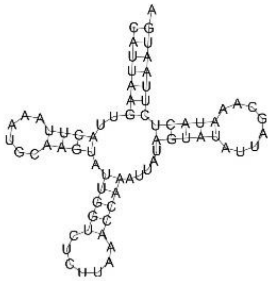

tRNA-K

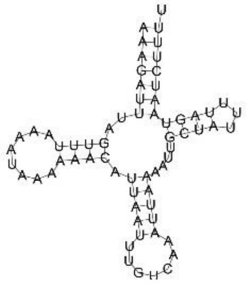

tRNA-D

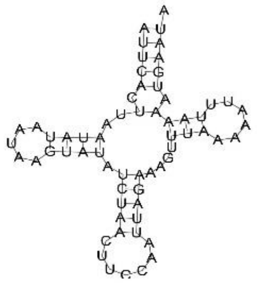

tRNA-G

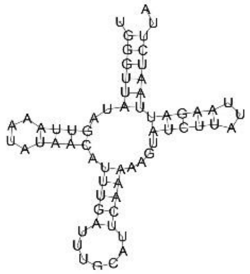

tRNA-A

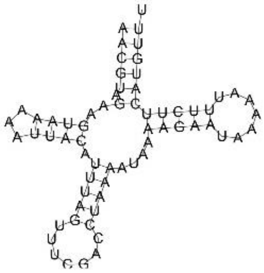

tRNA-R

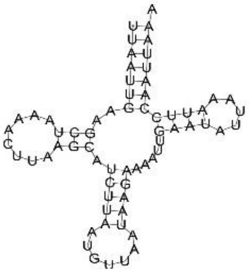

tRNA-N

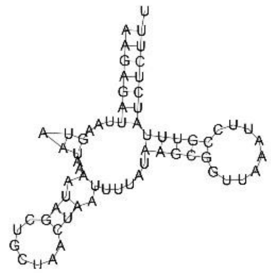

tRNA-S1

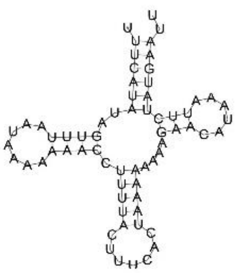

tRNA-E

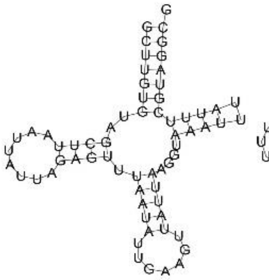

tRNA-F

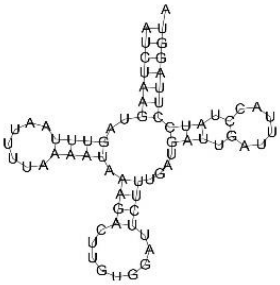

tRNA-H

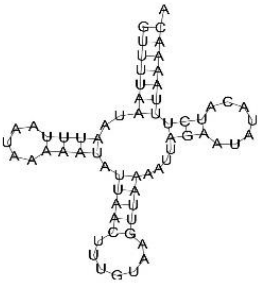

tRNA-T

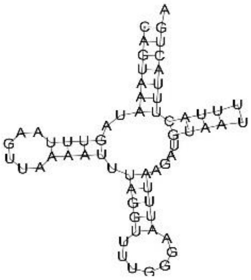

tRNA-P

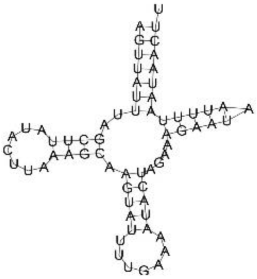

tRNA-S2

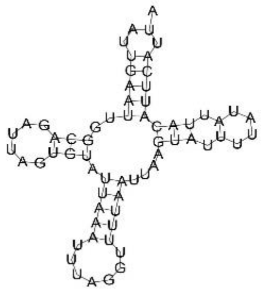

tRNA-L1

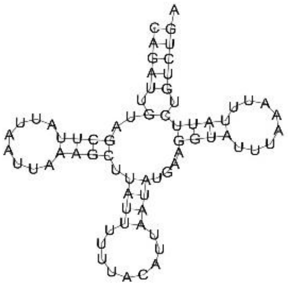

tRNA-V

(c) *Sahlbergotettix salicicola.*

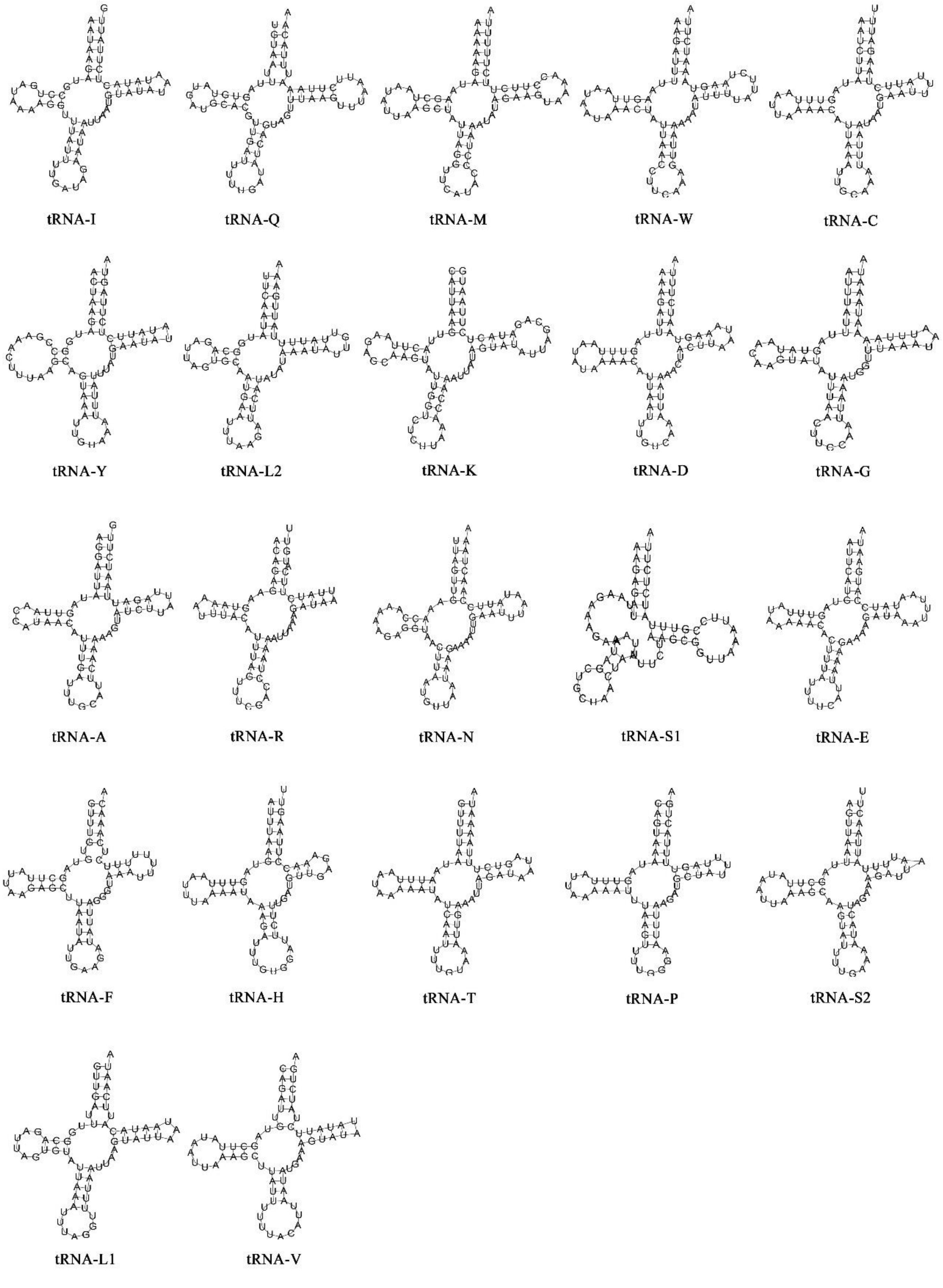

(d) *Podulmorinus opacus*.

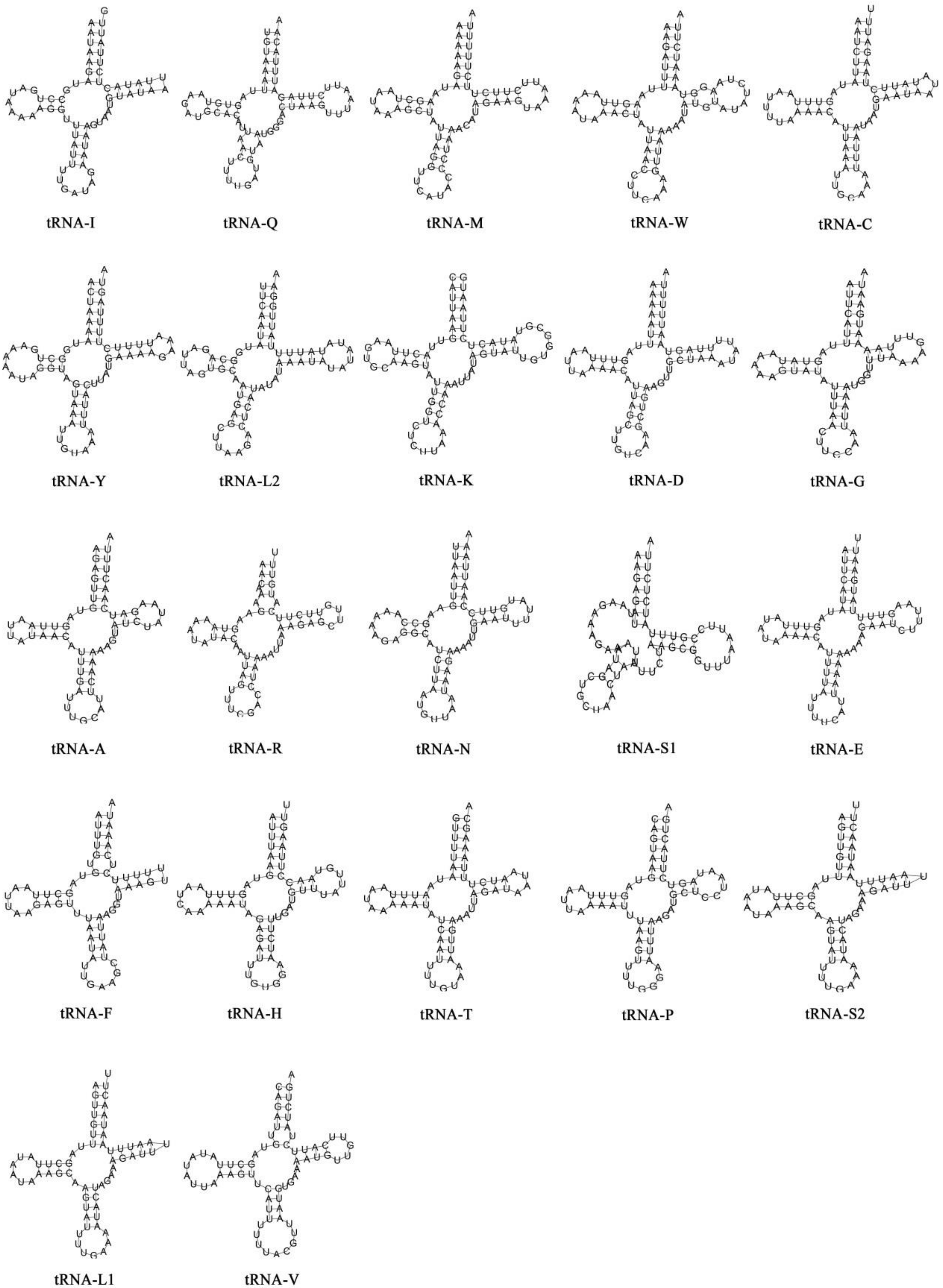

(e) *Podulmorinus consimilis*.

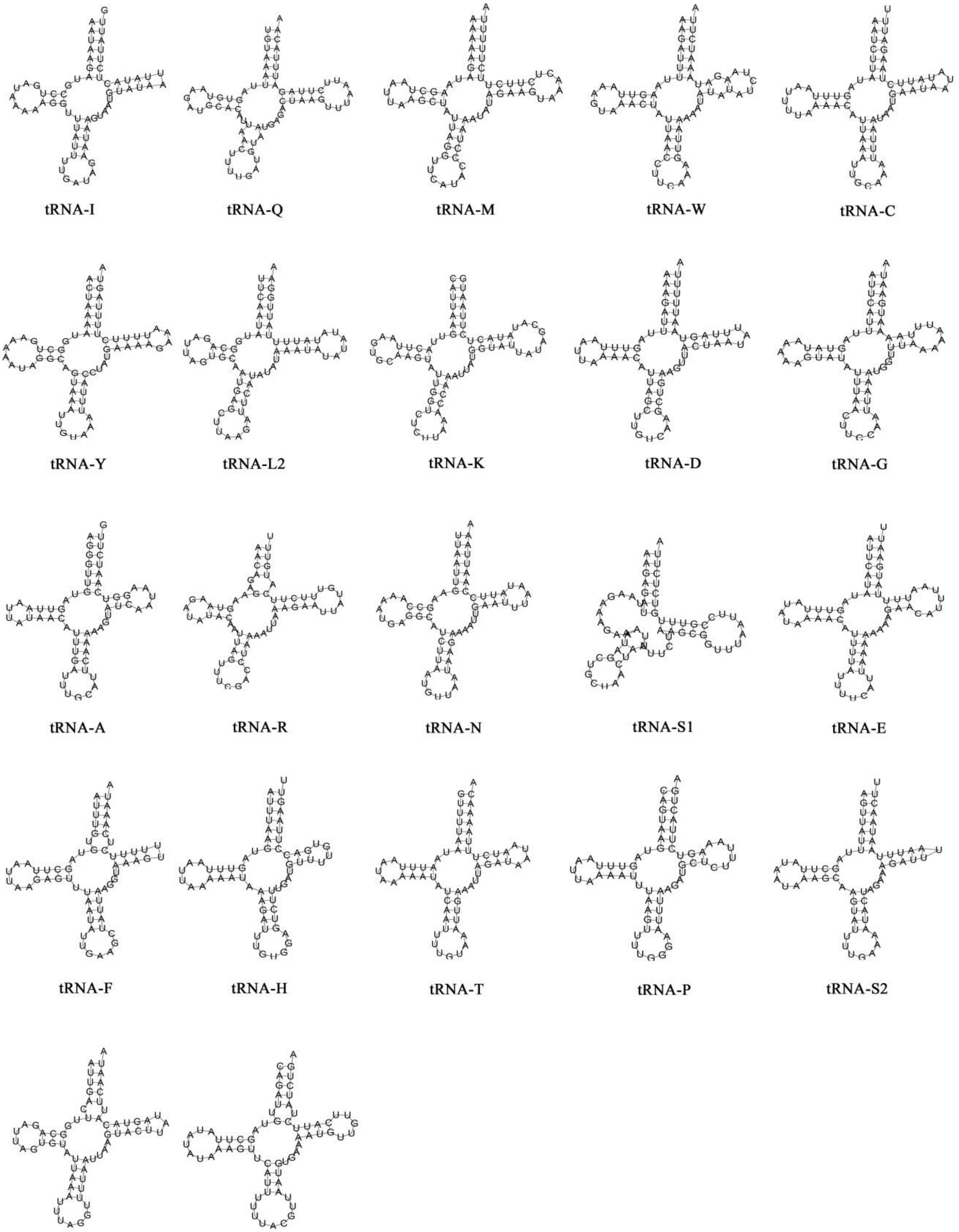

Supplement: Supplementary file 1 [file genes-13-02000-s001.zip › Figure S1.pdf]
