# Supplementary material for: Complete Mitogenome Analysis of Five Leafhopper Species of Idiocerini (Hemiptera: Cicadellidae)
Source: Genes (Basel). 2022 Nov 1;13(11):2000. doi: 10.3390/genes13112000 (PMC9690763; doi:10.3390/genes13112000)
Supplement: Supplementary file 1 [file genes-13-02000-s001.zip › Table S1.pdf]

Table S1. Organization of mitochondrial genomes of the tribe Idiocerini

Name	Direction	Location	Size(bp)	Start	Stop	Anticodon	IN
		Nabicerus dentimus/ *Neoamritatus* sp./ *Sahlbergotettix salicicola*/*Podulmorinus opacus*/*Podulmorinus consimilis*					
tRNA-I	F/F/F/F/F	1-65/1-65/1-65/1-65/1-65	65/65/65/65/65			GAT/31-33GAT/31-33GAT/31-33GAT/31-33GAT	-2/-2/-2/-2/-2
tRNA-Q	R/R/R/R/R	63-130/63-131/63-131/63-131/63-131	68/69/69/69/69			TTG/99-101TTG/99-101TTG/93-95TTG/99-101TTG	3/6/3/-1/-1
tRNA-M	F/F/F/F/F	134-201/138-205/135-204/131-198/131-198	68/68/70/69/68			CAT/168-170CAT/167-169CAT/161-163CAT/161-163CAT	0/0/0/0/0
nad2	F/F/F/F/F	202-1,176/206-1,180/205-1,179/199-1,173/199-1,173	975/975/975/975/975	ATT/ATT/ATT/ATC/ATC	TAA/TAA/TAA/TAA/TAA		-1/2/-1/-1/-1
tRNA-W	F/F/F/F/F	1,175-1,242/1,183-1,247/1,178-1,245/1,172-1,239/1172-1237	68/65/68/68/66			TCA/1,213-1,215TCA/1,210-1212TCA/1,203-1,205TCA/1,203-1,205TCA	-7/-7/-7/-7/-7
tRNA-C	F/F/F/F/F	1,235-1,297/1,240-1,302/1,238-1,300/1,232-1,298/1,230-1,296	63/63/63/67/67			GCA/1,270-1,272GCA/1,268-1,270GCA/1,264-1,266GCA/1,262-1,264GCA	7/4/2/7/7
tRNA-Y	F/F/F/F/F	1,305-1,372/1,307-1,373/1,303-1,368/1,306-1,372/1,304-1,370	68/67/66/67/67			GTA/1,339-1,341GTA/1,334-1,336GTA/1,339-1,341GTA/1,337-1,339GTA	11/7/12/7/7
cox1	F/F/F/F/F	1,384-2,918/1,381-2,916/1,381-2,888/1,380-2,915/1,378-2,912	1,535/1,536/1,508/1,536/1536	ATG/ATG/ATG/ATG/ATG	TA-/TAA/TA-/TAA/TA-		0/0/27/3/0

tRNA-L2	F/F/F/F/F	2,919-2,985/2,917-2,982/2,916-2,980/2,919-2,985/2,913-2,979	67/66/66/67/67			TAA/2,946-2,948TAA/2,945-2,947TAA/2,948-2,950TAA/2,942-2,944TAA	0/0/0/0/0
cox2	F/F/F/F/F	2,986-3,661/2,983-3,661/2,981-3,659/2,986-3,664/2,908-3,658	676/679/679/679/679	ATT/ATC/ATT/ATT/ATT	T--/ T--/T--/T--/T--		3/0/0/0/0
tRNA-K	F/F/F/F/F	3,665-3,735/3,662-3,733/3,660-3,730/3,665-3,735/3,659-3,729	71/72/71/71/71			CTT/3,693-3,695CTT/3,691-3,693CTT/3,696-3,698CTT/3,690,3,692CTT	0/0/0/0/0
tRNA-D	F/F/F/F/F	3,736-3,799/3,734-3,799/3,731-3,795/3,736-3,802/3,730-3,794	64/66/65/67/65			GTC/3,767-3,769GTC/3,762-3,764GTC/3,766-3,768GTC/3,760-3,762GTC	0/-1/0/0/0
atp8	F/F/F/F/F	3,800-3,952/3,799-3,951/3,796-3,948/3,803-3,955/3,795-3,947	153/153/153/153/153	TTG/TTG/TTG/TTG	TAA/TAA/TAA/TAA/TAA		-3/-3/-3/-6-6
atp6	F/F/F/F/F	3,949-4,602/3,948-4,601/3,945-4,598/3,949-4,605/3,941-4,597	654/654/654/657/657	ATA/TAA/ATA/ATG/ATG	TAA/TAA/TAA/TAA/TAA		1/0/5/1/1
cox3	F/F/F/F/F	4,604-5,383/4,602-5,379/4,604-5,383/4,607-5,386/4,599-5,378	780/778/780/780/780	ATG/ATG/ATG/ATG/ATG	TAA/T--/TAA/TAA/TAA		4/3/-1/0/-1
tRNA-G	F/F/F/F/F	5,388-5,452/5,383-5,446/5,383-5,447/5,386-5,449/5,378-5,441	65/64/65/64/64			TCC/5,413-5,415TCC/5,413-5,415TCC/5,416-5,418TCC/5,408-5,410TCC	0/0/0/0/0

nad3	F/F/F/F/F	5,453-5,806/5,447-5,800/5,448-5,801/5,450-5,803/5,442-5,795	354/354/354/354/354	ATT/ATA/ATC/ATT/ATT	TAA/TAA/TAG/TAA/TAA		-1/7/-1/-1/-1
tRNA-A	F/F/F/F/F	5,806-5,867/5,808-5,871/5,800-5,862/5,803-5,864/5,795-5,856	62/64/63/62/62			TGC/5,837-5,839TGC/5,829-5,831TGC/5,833-5,835TGC/5,824-5,826TGC	0/-1/0/5/5
tRNA-R	F/F/F/F/F	5,868-5,930/5,871-5,936/5,863-5,923/5,870-5,932/5,862-5,926	63/66/61/63/65			TCG/5,899-5,901TCG/5,890-5,892TCG/5,898-5,900TCG/5,890-5,892TCG	0/-1/0/-1/-1
tRNA-N	F/F/F/F/F	5,931-5,997/5,936-6,002/5,924-5,989/5,932-5,997/5,926-5,991	67/67/66/66/66			GTT/5,967-5,969GTT/5,954-5,956GTT/5,962-5,964GTT/5,956-5,958GTT	-1/-1/-1//-1/-1
tRNA-S1	F/F/F/F/F	5,997-6,062/6,002-6,067/5,989-6,054/5,997-6,062/5,991-6,056	66/66/66/66/66			GCT/6,026-6,028GCT/6,013-6,015GCT/6,021-6,023GCT/6,015-6,017GCT	4/4/1/0/0
tRNA-E	F/F/F/F/F	6,067-6,133/6,072-6,137/6,056-6,121/6,063-6,128/6,057-6,121	67/66/66/66/65			TTC/6,104-6,106TTC/6,085-6,087TTC/6,093-6,095TTC/6,087-6,089TTC	0/1/3/-1/-1
tRNA-F	R/R/R/R/R	6,132-6,193/6,139-6,203/6,125-6,187/6,128-6,190/6,121-6,183	62/65/63/63/63			GAA/6,168-6,170GAA/6,155-6,157GAA/6,157-6,159GAA/6,150-6,152	0/0/0/0/0
nad5	R/R/R/R/R	6,194-7,865/6,204-7,875/6,188-7,859/6,191-7,862/6,184-7,855	1,672/1,672/1,672/1,672/1,672	TTG/ATG/TTG/TTG/TTG	T--/T--/T--/T--/T--		0/1/0/0/0

tRNA-H	R/R/R/R/R	7,866-7,929/7,877-7,946/7,860-7,922/7,863-7,927/7,856-7,918	64/70/63/65/63			GTG/7,912-7,914GTG/7,889-7,891GTG/7,894-7,896GTG/7,885-7,887GTG	-1/9/0/-1/-1
nad4	R/R/R/R/R	7,928-9,241/7,956-9,258/7,923-9,231/7,927-9,228/7,918-9,219	1,314/1,303/1,309/1,302/1,302	ATT/ATA/ATT/ATA/ATA	TAA/T--/T--/TAA/TAA		-12/-3/-12/-1
nad4l	R/R/R/R/R	9,229-9,507/9,255-9,533/9,219-9,497/9,228-9,506/9,219-9,497	279/279/279/279/279	ATG/ATG/ATG/ATA/ATG	TAA/TAA/TAA/TAA/TAA		2/2/2/2/2
tRNA-T	F/F/F/F/F	9,510-9,572/9,536-9,600/9,500-9,563/9,509-9,572/9,500-9,563	63/65/64/64/64			TGT/9,566-9,568TGT/9,530-9,532TGT/9,539-9,541TGT/9,630-9,532TGT	0/0/0/0/0
tRNA-P	R/R/R/R/R	9,573-9,636/9,601-9,663/9,564-9,626/9,573-9,636/9,564-9,627	64/63/63/64/64			TGG/9,630-9,632TGG/9,634-9,636TGG/9,504-9,506TGG/9,596-9,598TGG	2/2/2/2/2
nad6	F/F/F/F/F	9,639-10,124/9,666-10,151/9,629-10,114/9,639-10,124/9,630-10,115	486/486/486/486/486	ATC/ATA/ATC/ATT/ATT	TAA/TAA/TAA/TAA/TAA		1/-1/-1/-1/-1
cob	F/F/F/F/F	10,124-11,260/10,151-11,287/10,114-11,250/10,124-11,263/10,115-11,254	1,137/1,137/1,137/1,137/1,140	ATG/ATG/ATG/ATG/ATG	TAA/TAA/TAA/TAA/TAA		-1/3/3//-1-1
tRNA-S2	F/F/F/F/F	11,260-11,324/11,291-11,355/11,254-11,318/11,263-11,326/11,254-11,317	65/65/65/64/64			TGA/11,323-11,325TGA/11,286-11,288TGA/11,294-11,296TGA/11,283-11,285TGA	1/2/2/1/1

nad1	R/R/R/R/R	11,326-12,256/11,358-12,288/11,321-12,251/11,328-12,259/11,319-12,250	931/931/931/932/932	ATG/ATT/ATT/ATT/ATT	T--/ T--/ T--/TA-/TA-		0/0/0/0/0
tRNA-L1	R/R/R/R/R	12,257-12,320/12,289-12,353/12,252-12,315/12,206-12,323/12,251-12,314	64/65/64/64/64			TAG/12,322-12,324TAG/12,284-12,286TAG/12,292-12,294TAG/12,281-12,283TAG	-16/5/17/-1/81
16S rRNA	R/R/R/R/R	12,304-13,346/12,367-13,305/12,323-13,334/12,323-13,334/12,396-13,534	1,043/1,203/1,012/1,203/1,239				174/252/169/192/-14
tRNA-V	R/R/R/R/R	13,521-13,587/13,558-13,628/13,504-13,569/13,527-13,595/13.520-13,586	67/67/66/68/67			TAC/13,593-13,594TAC/13,535-13,537TAC/13,559-13,561TAC/13,552-13,554TAC	0/0/3/-22/0
12S rRNA	R/R/R/R/R	13,588-14,339/13,629-14,372/13,573-14,314/13,573-14,314/15,587-14,328	752/754/741/754/742				0/0/0/0/0
CR		14,340-14,815/14,373-15,044/14,315-14,733/14,315-14,815/14,329-14,825	476/672/419/501/497				
