# Supplementary material for: Complete Mitogenome Analysis of Five Leafhopper Species of Idiocerini (Hemiptera: Cicadellidae)
Source: Genes (Basel). 2022 Nov 1;13(11):2000. doi: 10.3390/genes13112000 (PMC9690763; doi:10.3390/genes13112000)
Supplement: Supplementary file 1 [file genes-13-02000-s001.zip › Table S2.pdf]

Table S2. Best-fit models and partitioning schemes selected by PartitionFinder 2

Partition names	Subset	sites	Best Model
atp6	1-654	654	GTR+I+G
atp8, nad6, na3d	655-804；11002-11493；7000-7353	996	GTR+I+G
cox1	805-2340	1536	TIM+I+G
cox2, cox3	2341-3018；3019-3795	1455	TIM+I+G
cob	3796-5091	1296	GTR+G
nad1	5092-6027	936	TVM+I+G
nad2	6028-6999	972	TVM+I+G
nad4l	7354-7782	429	GTR+I+G
nad4	7783-9120	1338	TVM+I+G
nad5	9121-11001	1881	GTR+G
