# Supplementary material for: Complete Mitogenome Analysis of Five Leafhopper Species of Idiocerini (Hemiptera: Cicadellidae)
Source: Genes (Basel). 2022 Nov 1;13(11):2000. doi: 10.3390/genes13112000 (PMC9690763; doi:10.3390/genes13112000)
Supplement: Supplementary file 1 [file genes-13-02000-s001.zip › Table S3.pdf]

Table S3. List of complete mitogenomes of leafhoppers in the family Cicadellidae retrieved from GenBank

Organism	Family	Genus	Length (bp)	GenBank accession number
Abrus daozhenensis	Cicadellidae	*Abrus*	16391	MZ274046
Abrus expansivus	Cicadellidae	*Abrus*	15904	MK033020
Abrus yunshanensis	Cicadellidae	*Abrus*	16298	MZ274047
Aconurella prolixa	Cicadellidae	*Aconurella*	15222	MZ433366
Agnesiella kamala	Cicadellidae	*Agnesiella*	15209	MZ457327
Agnesiella Roxana	Cicadellidae	*Agnesiella*	15901	MZ457328
Aguriahana digitate	Cicadellidae	*Aguriahana*	15854	MZ457330
Alebroides salicis	Cicadellidae	*Alebroides*	15890	MZ014449
Atkinsoniella curvata	Cicadellidae	*Atkinsoniella*	15988	OL677864
Atkinsoniella flavipenna	Cicadellidae	*Atkinsoniella*	15671	OL677865
Atkinsoniella grahami	Cicadellidae	*Atkinsoniella*	15621	MW533712
Atkinsoniella heiyuana	Cicadellidae	*Atkinsoniella*	16368	MG813482
Atkinsoniella longiuscula	Cicadellidae	*Atkinsoniella*	15815	OL677866
Atkinsoniella nigrita	Cicadellidae	*Atkinsoniella*	16011	ON009029
Atkinsoniella thalia	Cicadellidae	*Atkinsoniella*	15034	OL677867
Atkinsoniella thaloidea	Cicadellidae	*Atkinsoniella*	15571	OL677868
Atkinsoniella tiani	Cicadellidae	*Atkinsoniella*	15841	OL677869
Atkinsoniella uniguttata	Cicadellidae	*Atkinsoniella*	15784	OL677870
Atkinsoniella warpa	Cicadellidae	*Atkinsoniella*	15852	OL677871
Atkinsoniella wui	Cicadellidae	*Atkinsoniella*	15159	OL677872
Atkinsoniella xanthonota	Cicadellidae	*Atkinsoniella*	15472	MT596736
Atkinsoniella yunnanana	Cicadellidae	*Atkinsoniella*	15875	OL677874
Atkinsoniella zizhongi	Cicadellidae	*Atkinsoniella*	16483	ON457601
Balala fujiana	Cicadellidae	*Balala*	16221	MW218661
Balala lui	Cicadellidae	*Balala*	15558	MG813488
Batracomorphus lateprocessus	Cicadellidae	*Batracomorphus*	15356	MG813489
Bolanusoides shaanxiensis	Cicadellidae	*Bolanusoides*	15274	MN661136
Bothrogonia ferruginea	Cicadellidae	*Bothrogonia*	15262	KU167550
Bothrogonia qiongana	Cicadellidae	*Bothrogonia*	15788	MT500855
Bothrogonia shuana	Cicadellidae	*Bothrogonia*	15229	MT500856
Bothrogonia tongmaiana	Cicadellidae	*Bothrogonia*	15539	MT500857
Bothrogonia yunana	Cicadellidae	*Bothrogonia*	15585	MT500858
Carinata rufipenna	Cicadellidae	*Carinata*	15261	MN227165
Cassianeura bimaculata	Cicadellidae	*Cassianeura*	14597	MT985381
Cassianeura cassiae	Cicadellidae	*Cassianeura*	15423	MT985380
Changbaninus pleiospicules	Cicadellidae	*Changbaninus*	15527	MT332317
Chudania hellerina	Cicadellidae	*Chudania*	15044	MN227164
Cicadella viridis	Cicadellidae	*Cicadella*	15891	MK335936
Cofana yasumatsui	Cicadellidae	*Cofana*	15019	MN793964
Concaveplana rufolineata	Cicadellidae	*Concaveplana*	15240	MN227168
Dikraneura zlata	Cicadellidae	*Dikraneura*	15330	MZ014450
Dikraneurini sp. SL-2021a	Cicadellidae	*Dikraneurini*	15306	MZ014451
Doratura homophyla	Cicadellidae	*Doratura*	16269	OK105076
Doratura stylata	Cicadellidae	*Doratura*	16054	OK105077
Drabescoides nuchalis	Cicadellidae	*Drabescoides*	15309	KR349344
Drabescus ineffectus	Cicadellidae	*Drabescus*	15744	MT527188
Durgades nigropicta	Cicadellidae	*Durgades*	15974	KY123686
Elbelus tripunctatus	Cicadellidae	*Elbelus*	15308	MZ014452
Empoasca serrata	Cicadellidae	*Empoasca*	15131	MZ014453
Empoasca vitis	Cicadellidae	*Empoasca*	15154	MG813494
Empoascanara dwalata	Cicadellidae	*Empoascanara*	15271	MT350235
Empoascanara gracilis	Cicadellidae	*Empoascanara*	14627	MT576649
Empoascanara sipra	Cicadellidae	*Empoascanara*	14827	MN604278
Empoascanara wengangensis	Cicadellidae	*Empoascanara*	14830	MT445764
Eupteryx adspersa	Cicadellidae	*Eupteryx*	15178	MZ014454
Eupteryx gracilirama	Cicadellidae	*Eupteryx*	17173	MT594485

Eupteryx minuscula	Cicadellidae	*Eupteryx*	16944	MN910279
Eurhadina acapitata	Cicadellidae	*Eurhadina*	15419	MZ457331
Eurhadina dongwolensis	Cicadellidae	*Eurhadina*	15708	MZ457332
Eurhadina fusca	Cicadellidae	*Eurhadina*	15302	MZ983367
Eurhadina jarrayi	Cicadellidae	*Eurhadina*	15332	MZ014455
Evacanthus acuminatus	Cicadellidae	*Evacanthus*	14793	MK948205
Evacanthus danmainus	Cicadellidae	*Evacanthus*	15343	MN227166
Evacanthus heimianus	Cicadellidae	*Evacanthus*	15806	MG813486
Exitianus nanus	Cicadellidae	*Exitianus*	15714	OK105078
Fieberiella septentrionalis	Cicadellidae	*Fieberiella*	16175	MW078430
Ghauriana sinensis	Cicadellidae	*Ghauriana*	15491	MN699874
Gunungidia aurantiifasciata	Cicadellidae	*Gunungidia*	15669	MT622821
Hylica paradoxa	Cicadellidae	*Hylica*	14762	MW218660
Idiocerus herrichii	Cicadellidae	*Idiocerus*	15489	MN935487
Idiocerus salicis	Cicadellidae	*Idiocerus*	16436	MG813490
Idioscopus clypealis	Cicadellidae	*Idioscopus*	15393	NC_039642
Idioscopus nitidulus	Cicadellidae	*Idioscopus*	15287	NC_029203
Jacobiasca formosana	Cicadellidae	*Jacobiasca*	16353	MW429482
Japanagallia spinosa	Cicadellidae	*Japanagallia*	15655	KY123687
Japananus hyalinus	Cicadellidae	*Japananus*	15364	KY129954
Kalasha nativa	Cicadellidae	*Kalasha*	15716	MW218662
Kaukania anser	Cicadellidae	*Kaukania*	15345	MZ014456
Kolla paulula	Cicadellidae	*Kolla*	14995	MW542170
Koreocerus koreanus	Cicadellidae	*Koreocerus*	15594	MW916101
Krisna concava	Cicadellidae	*Krisna*	14304	MN577635
Krisna rufimarginata	Cicadellidae	*Krisna*	14724	MN577636
Kusala populi	Cicadellidae	*Kusala*	15402	OM066070
Ledra auditura	Cicadellidae	*Ledra*	16094	MK387845
Ledra trigona	Cicadellidae	*Ledra*	16094	MG813491
Limassolla emmrichi	Cicadellidae	*Limassolla*	14677	MW272458
Limassolla lingchuanensis	Cicadellidae	*Limassolla*	15716	MN605256
Limassolla sp. XZ-2020	Cicadellidae	*Limassolla*	17053	MT683892
Macropsis notata	Cicadellidae	*Macropsis*	16323	KX010420
Macrosteles quadrilineatus	Cicadellidae	*Macrosteles*	16626	KY645960
Macrosteles quadrimaculatus	Cicadellidae	*Macrosteles*	15734	MG727894
Maiestas dorsalis	Cicadellidae	*Maiestas*	15352	KX786285
Matsumurasca onukii	Cicadellidae	*Matsumurasca*	15167	MG190360
Membranacea stenoprocessa	Cicadellidae	*Membranacea*	14717	MW426467
Mesargus serrata	Cicadellidae	*Mesargus*	15399	MG813495
Metidiocerus impressifrons	Cicadellidae	*Metidiocerus*	16426	MW963341
Metidiocerus sp.	Cicadellidae	*Metidiocerus*	15079	MT554451
Michalowskiya breviprocessa	Cicadellidae	*Michalowskiya*	15591	MW264489
Mileewa albovittata	Cicadellidae	*Mileewa*	15079	MK138358
Mileewa margheritae	Cicadellidae	*Mileewa*	15375	MT483998
Mileewa ponta	Cicadellidae	*Mileewa*	15999	MT497465
Mileewa rufivena	Cicadellidae	*Mileewa*	15837	MZ326689
Mimotettix multispinosus	Cicadellidae	*Mimotettix*	15754	MT332321
Mitjaevia bifurcata	Cicadellidae	*Mitjaevia*	16813	OK448488
Mitjaevia diana	Cicadellidae	*Mitjaevia*	16589	OK448489
Mitjaevia dworakowskae	Cicadellidae	*Mitjaevia*	16399	MT981880
Mitjaevia protuberanta	Cicadellidae	*Mitjaevia*	15472	MN627216
Mitjaevia shibingensis	Cicadellidae	*Mitjaevia*	15788	MT981879
Mukaria splendida	Cicadellidae	*Mukaria*	16711	MG813485
Nacolus tuberculatus	Cicadellidae	*Nacolus*	15737	MW218663
Nephotettix cincticeps	Cicadellidae	*Nephotettix*	14805	KP749836
Nephotettix malayanus	Cicadellidae	*Nephotettix*	15462	OK105079
Nephotettix nigropictus	Cicadellidae	*Nephotettix*	15280	OK105080
Nephotettix virescens	Cicadellidae	*Nephotettix*	15810	OK105081
Nesophrosyne haleakala	Cicadellidae	*Nesophrosyne*	15513	ON135509
Nesophrosyne kanawao	Cicadellidae	*Nesophrosyne*	15178	ON135519

Nesophrosyne makaihe	Cicadellidae	*Nesophrosyne*	15679	ON135524
Nesophrosyne maritima	Cicadellidae	*Nesophrosyne*	15579	ON135520
Nesophrosyne montium	Cicadellidae	*Nesophrosyne*	15194	ON135508
Nesophrosyne ogradyi	Cicadellidae	*Nesophrosyne*	16349	ON135505
Nesophrosyne ponapona	Cicadellidae	*Nesophrosyne*	15138	ON135522
Nesophrosyne sp. 246 GMB-2012	Cicadellidae	*Nesophrosyne*	15051	ON135521
Olidiana longisticka	Cicadellidae	*Olidiana*	15993	MN780582
Olidiana obliquea	Cicadellidae	*Olidiana*	15312	MN780583
Olidiana ritcheri	Cicadellidae	*Olidiana*	15372	MN780584
Olidiana ritcheriina	Cicadellidae	*Olidiana*	15205	MN780581
Olidiana tongmaiensis	Cicadellidae	*Olidiana*	15363	MN780585
Oncopsis nigrofasciata	Cicadellidae	*Oncopsis*	15927	MG813492
Orosius sp.	Cicadellidae	*Orosius*	15374	ON135523
Paraahimia luodianensis	Cicadellidae	*Paraahimia*	16497	MN901449
Paradorydium reflexanum	Cicadellidae	*Paradorydium*	15661	MG813487
Paralaevicephalus gracilipenis	Cicadellidae	*Paralaevicephalus*	16114	MK450366
Paramacrosteles nigromaculatus	Cicadellidae	*Paramacrosteles*	15011	MK234840
Parascaphoidella transversa	Cicadellidae	*Parascaphoidella*	16011	MT332318
Parathailocyba orla	Cicadellidae	*Parathailocyba*	15382	MN894531
Parocerus laurifoliae	Cicadellidae	*Idiocerus*	16811	MH433622
Penthimia melanocephala	Cicadellidae	*Penthimia*	15308	MT768010
Petalocephala chlorophana	Cicadellidae	*Petalocephala*	14927	MT610899
Petalocephala eurglobata	Cicadellidae	*Petalocephala*	14834	MW018817
Petalocephala gongshanensis	Cicadellidae	*Petalocephala*	14969	MW018818
Phlogotettix cyclops	Cicadellidae	*Phlogotettix*	15078	MT332320
Phlogotettix monozoneus	Cicadellidae	*Phlogotettix*	15201	MT332315
Phlogothamnus polymaculatus	Cicadellidae	*Phlogothamnus*	14905	MT332322
Populicerus confusus	Cicadellidae	*Populicerus*	16395	MT341642
Populicerus populi	Cicadellidae	*Populicerus*	16494	MH492318
Reticuluma hamata	Cicadellidae	*Reticuluma*	15190	MN922303
Roxasellana stellata	Cicadellidae	*Roxasellana*	15361	MT527187
Scaphoideus maai	Cicadellidae	*Scaphoideus*	15188	KY817243
Scaphoideus maculatus	Cicadellidae	*Scaphoideus*	15486	MT332316
Scaphoideus nigrivalveus	Cicadellidae	*Scaphoideus*	15235	KY817244
Scaphoideus varius	Cicadellidae	*Scaphoideus*	15207	KY817245
Signoretia aureola	Cicadellidae	*Signoretia*	15547	MG813493
Taharana fasciana	Cicadellidae	*Taharana*	15161	KY886913
Tituria pyramidata	Cicadellidae	*Tituria*	15331	MN920440
Tituria sagittata	Cicadellidae	*Tituria*	14918	MT610900
Trifida elongata	Cicadellidae	*Trifida*	14924	MT153579
Trocnadella arisana	Cicadellidae	*Trocnadella*	15131	KU748516
Ujna puerana	Cicadellidae	*Ujna*	14838	MZ326688
Yangisunda tiani	Cicadellidae	*Yangisunda*	15190	MZ014459
Yanocephalus yanonis	Cicadellidae	*Yanocephalus*	15623	KY039113
Ziczacella heptapotamica	Cicadellidae	*Ziczacella*	15334	OM066071
Zyginella minuta	Cicadellidae	*Zyginella*	15544	MT488436
