# Supplementary material for: Complete Mitogenome Analysis of Five Leafhopper Species of Idiocerini (Hemiptera: Cicadellidae)
Source: Genes (Basel). 2022 Nov 1;13(11):2000. doi: 10.3390/genes13112000 (PMC9690763; doi:10.3390/genes13112000)
Supplement: Supplementary file 1 [file genes-13-02000-s001.zip › Table S4.pdf]

Table S4 Tandem repeats in the mitochondrial control region of 14 species in the tribe Idiocerini

(1)*Idiocerus herrichii*

Unit	Position		Length	Copy	5'–3' motif sequence
	From	To	(bp)	number	
TR1	89	159	27	2.6	AAATTTATATAAAATAATTTTAAACAA
TR2	173	210	20	2.0	TTAAATTAAATAATAAATAT
TR3	550	740	18	10.1	ATATAATTAATAATAATA

(2) *Populicerus confusus*

Unit	Position		Length	Copy number	5'–3' motif sequence
	From	To	(bp)		
TR1	60	309	80	3.1	TTTAAATAATTTGACTTAGTAGGGGCTAAGTAATTATAAATTTATGAAATGAATGGTTAC TAACTAATTGAATTTCTAAT
TR2	433	816	80	4.8	TAAGTAATTATAAATTTATGAAATGAATGGTTACTAACTAATTGAATTTCTAATTTTAAA TAATTTGACTTAGTAGGGGC
TR3	939	1242	80	3.8	TAAGTAATTATAAATTTATGAAATGAATGGTTACTAACTAATTGAATTTCTAATTTTAAA TAATTTGACTTAGTAGGGGC
TR4	1770	1835	18	3.7	ATTAATTAATTATATAGT
TR5	1899	1950	17	3.4	ATATATTAATTATATAT

(3)*Metidiocerus sp.*

Unit	Position		Length	Copy numbe	5'–3' motif sequence
	From	To	(bp)		
TR1	377	426	21	2.5	TATATTAATATAGTAAATAAA

(4)*Rhytidodus viridiflavus*

Unit	Position		Length	Copy number	5'–3' motif sequence
	From	To	(bp)		
TR1	249	289	18	2.3	TAATATAATAAATTAATA
TR2	344	658	22	14.9	ATATATATATAATAAATATAAT
TR3	763	799	19	2.1	ATAAATATAATAAATTAAT
TR4	857	910	9	6.2	TAAATATAA
TR5	1407	1451	17	2.6	TTATCAATAAACTAATA

(5) *Sahlbergotettix salicicola*

Unit	Position		Length	Copy number	5'–3' motif sequence
	From	To	(bp)		
TR1	63	241	51	3.5	ACTCAATAAATTTTATTGAAATAAGAAATAGTAACTAATAAGGCAAACATC
TR2	248	327	28	2.9	ATTAATATATATATATTTATATATTATA

(6)*Podulmorinus consimilis*

Unit	Position		Length	Copy number	5'–3' motif sequence
	From	To	(bp)		
TR1	245	320	15	5.1	ATTAATTATATATTT
TR2	389	424	15	2.4	ATTAATTATATATTT

(7) *Koreocerus koreanus*

Unit	Position		Length	Copy number	5'–3' motif sequence
	From	To	(bp)		
TR1	360	868	205	2.4	AAATAAAATCATAGTATTTATATCCATTTAATTAGGGGTTAAATAGAGATAAATAATAAATTTTATAGTTCATTAAAGTTAATTTAACTATTCAATTTAATTGTATAATTTATTATTGATTTAAGATTAGAATGTAAATTATTTAACTATATAATCCTACAAACCTATTTGTTATTAAAACTAAAAGAGGTTTGT
TR2	1148	1203	28	2.0	ATATTATAATTAGTATATAATAATTATT

(8)*Metidiocerus impressifrons*

Unit	Position		Length	Copy number	5'–3' motif sequence
	From	To	(bp)		
TR1	311	370	14	4.4	TATTAATATATATA
TR2	425	484	14	4.4	TATTAATATATATA
TR3	539	598	14	4.4	TATTAATATATATA
TR4	653	712	14	4.4	TATTAATATATATA
TR5	767	826	14	4.4	TATTAATATATATA
TR6	881	940	14	4.4	TATTAATATATATA
TR7	995	1054	14	4.4	TATTAATATATATA
TR8	1109	1168	14	4.4	TATTAATATATATA
TR9	1223	1282	14	4.4	TATTAATATATATA
TR10	1337	1396	14	4.4	TATTAATATATATA
TR11	1451	1510	14	4.4	TATTAATATATATA
TR12	1565	1624	14	4.4	TATTAATATATATA
TR13	1679	1738	14	4.4	TATTAATATATATA
TR14	1793	1852	14	4.4	TATTAATATATATA
TR15	1904	1969	15	4.7	TATTAATTATATATA

(9)*Idioscopus* sp.

Unit	Position		Length	Copy number	5'–3' motif sequence
	From	To	(bp)		
TR1	50	648	218	2.7	TCAAATTAAGGTTATGTTAAAATTAACTATAAACTGTAAGTAGATACACTATACTCCTACAAACCTCTTTTTTACTTAAATTAAAAGAGGTTTGTATATTTTATAGTTAAATGAACTTAATATTAAACTTAATTAGGGGTTAAGAATAATATTAAATATGTAGAATAACTATAATATACCCCCTTTTATATCTAA
TR2	805	870	18	3.8	TTATTTTATTATATTAAT

(10)*Idioscopus nitidulus*

Unit	Position		Length	Copy number	5'–3' motif sequence
	From	To	(bp)		
TR1	667	734	23	3.1	AATAATAATTATAATTATATATA

(11) *Idioscopus clypealis*

Unit	Position		Length	Copy number	5'–3' motif sequence
	From	To	(bp)		
TR1	35	673	152	4.2	TACTTTAATTAGGGGTTAAAGTATAAATTCTTCTTAATTAAGATAGTTATATAAGTGTAAT AGTA<br><br>GATAACTTTATAATTTACTCAAACCTCTTTTTTTCTAACTAGAAAAGAGGTTTGAATTAAA GATTAAGATTAATTTAAGTAGGCTTA

(12) *Idiocerus salicis*

Unit	Position		Length	Copy number	5'–3' motif sequence
	From	To	(bp)		
TR1	203	1576	131	10.4	TATTAACCTTTGACTTAAAGTGGATACATAAAGAATATCTATATAGAACTAATTTAGATG TATAT<br>TGTTACCATATTAATAATTCAAATTTTAACTACTTATCTAATAATGGGAGAATAGTATTTA AGTA<br>A
TR2	1675	1732	15	3.9	GTATTTATTAATTAA
TR3	1834	1882	23	2.1	ATTATTATTAATTCATTTATTTT

(13)*Idiocerus laurifoliae*

Unit	Position		Length	Copy number	5'–3' motif sequence
	From	To	(bp)		
TR1	34	77	17	2.8	TAATAAAACTAAAATAC
TR2	141	181	20	2.1	AAATAGAAATAGAATAACAA
TR3	211	272	15	3.8	TAAAATTAAATAAAT
TR4	363	619	81	3.2	AAAATAGATAAGTACCAACTACTTAAATAGAAATGAATAACAAAAATAGTAATAATAG GTCAATTCAACTAAACTCCATAG
TR5	662	1121	91	5.1	TAATAAAATTAGATTTATTAAGTATTATTTGTTATATCTTAAATATTAGTTATTAAAATA AACTT<br>AATTAGGGGTTAAGTTATATATTAAC
TR6	1479	1707	41	5.1	ATATTAGTTATTAAAATAAACTTAATTAGGGGTTAAGTTAT
TR7	2079	2109	15	2.0	TATTTATTTATATAT
TR8	2160	2292	13	9.8	TTATTTATATTAT
TR9	2334	2394	4.0	15	TTATTATATATTATT

(14) *Populicerus populi*

Unit	Position		Length	Copy number	5'–3' motif sequence
	From	To	(bp)		
TR1	59	465	80	5.1	AATTTAAAATAATTTAACTTAGTAGGGATTAAGGAATTATAAATTTATGAAATGAATGG<br>TTACTA<br>ACTAATTGAATTTCT
TR2	593	973	80	4.8	ACTAAGCAATTATAAATTTATGAAATGAATGGTTACTAACTAATTGAATTTCTAATTTA<br>AAATAA<br>TTTAACTTAGTAGGG
TR3	1101	1408	80	3.8	ACTAAGCAATTATAAATTTATGAAATGAATGGTTACTAACTAATTGAATTTCTAATTTA<br>AAATAA<br>TTTAACTTAGTAGGG
TR4	1985	2047	22	2.9	TTATTATTAATATTTATATGTA
