# Supplementary figures and images for: Complete Mitogenome Analysis of Five Leafhopper Species of Idiocerini (Hemiptera: Cicadellidae)
Source: Genes (Basel). 2022 Nov 1;13(11):2000. doi: 10.3390/genes13112000 (PMC9690763; doi:10.3390/genes13112000)

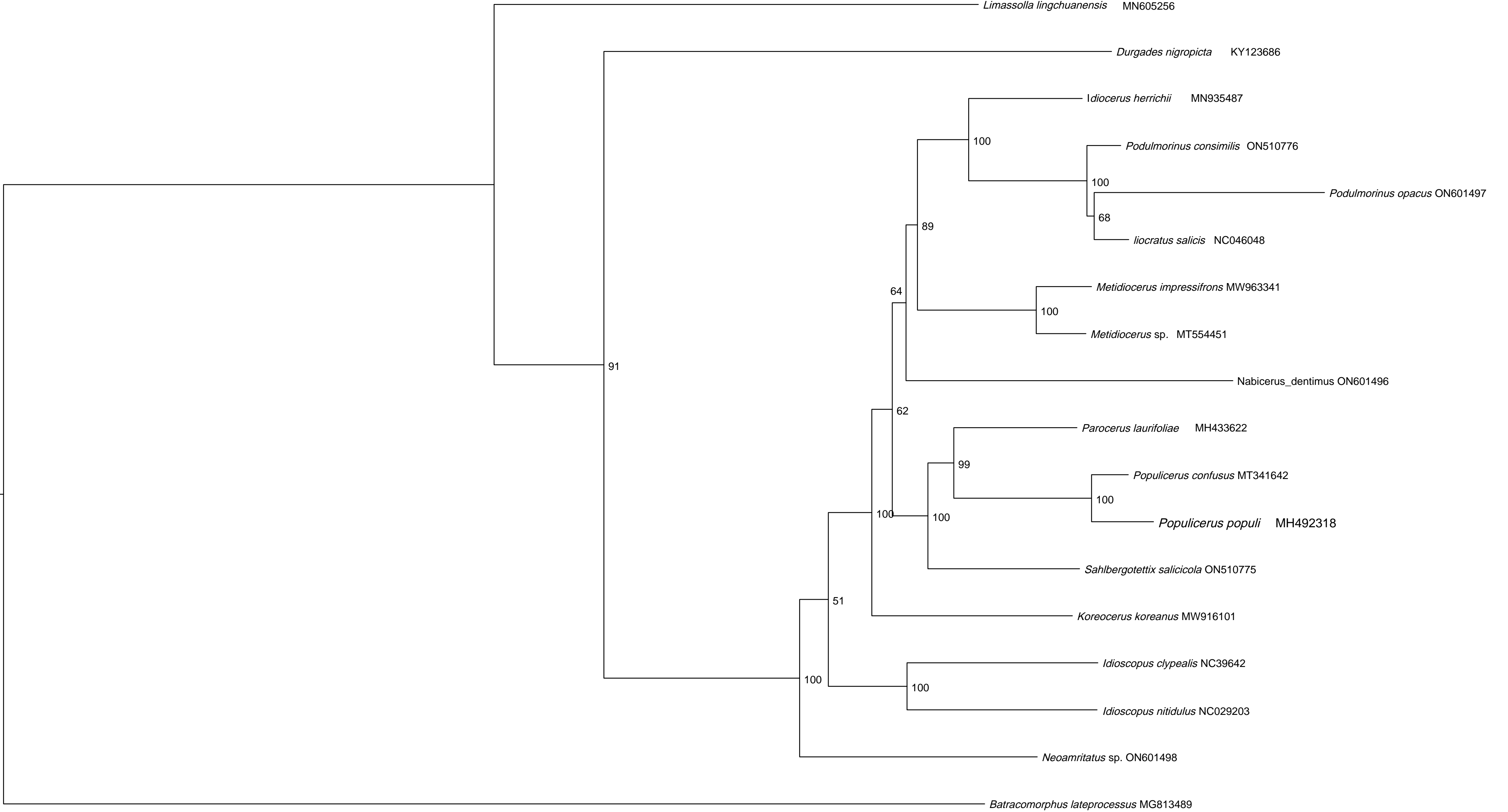

Supplement: Supplementary file 1 [file genes-13-02000-s001.zip › Figure S2.pdf]
